# A Principle for Describing and Verifying Brain Mechanisms Using Ongoing Activity

**DOI:** 10.3389/fncir.2017.00001

**Published:** 2017-01-24

**Authors:** David Eriksson

**Affiliations:** ^1^Center for Neuroscience, Albert Ludwig University of FreiburgFreiburg, Germany; ^2^BrainLinks-BrainTools, Albert Ludwig University of FreiburgFreiburg, Germany

**Keywords:** brain hypothesis, genesis of neuronal activity, neural input, ongoing activity, spontaneous activity, brain mechanisms

## Abstract

Not even the most informed scientist can setup a theory that takes all brain signals into account. A neuron not only receives neuronal short range and long range input from all over the brain but a neuron also receives input from the extracellular space, astrocytes and vasculature. Given this complexity, how does one describe and verify a typical brain mechanism *in vivo*? Common to most described mechanisms is that one focuses on how one specific input signal gives rise to the activity in a population of neurons. This can be an input from a brain area, a population of neurons or a specific cell type. All remaining inputs originating from all over the brain are lumped together into one background input. The division into two inputs is attractive since it can be used to quantify the relative importance of either input. Here we have chosen to extract the specific and the background input by means of recording and inhibiting the specific input. We summarize what it takes to estimate the two inputs on a single trial level. The inhibition should not only be strong but also fast and the specific input measurement has to be tailor-made to the inhibition. In essence, we suggest ways to control electrophysiological experiments *in vivo*. By applying those controls it may become possible to describe and verify many brain mechanisms, and it may also allow the study of the integration of spontaneous and ongoing activity, which in turn governs cognition and behavior.

## Introduction

**Figure 1:**
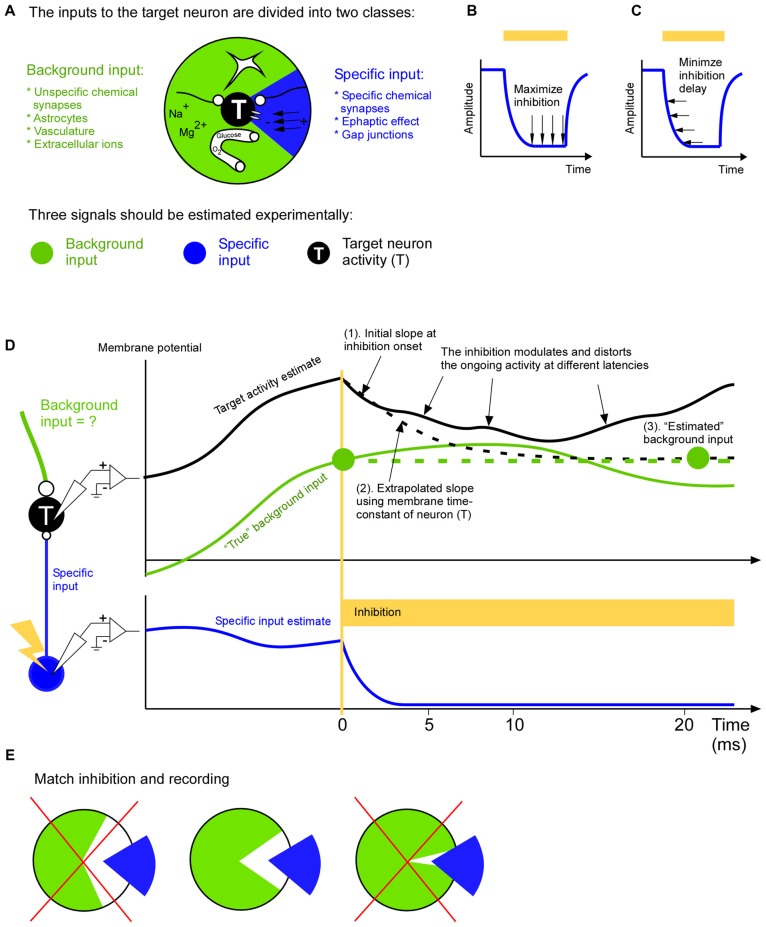
**A complete input mapping to a target population. (A)** The total input to the target neuron (T) is divided into a background input (*green*) and a specific input (*blue*). The specific input represents the signal from a neuronal cell-type, population or area of specific interest. The background input represents the remaining signals from astrocytes, long range and short range unspecific chemical synapses, vasculature and extracellular ions. **(B)** In order to be able to estimate the background input accurately the inhibition of the specific input should be close to complete. In two recent studies optogenetic inhibition has been reported to be around 90% (Reinhold et al., 2015; Li et al., 2016). **(C)** In order to be able to estimate the background input accurately the inhibition of the specific input should be fast. **(D)** To estimate the background input the specific input is inhibited. In this example the target membrane potential (*black*) is roughly the sum of the specific input (*blue*) and background input (*green*). The removal of the specific input will cause the target membrane potential to change towards the background input. After a few milliseconds this change will spread to neurons surrounding the target neuron. This modulation of the activity of the neighboring neurons will in turn feedback to the target neuron. This causes a growing distortion of the natural ongoing input. This cascade will continue to inter-areal neurons and astrocytes to name a few. Therefore the background measurement may be based on the initial change (*1*). Given that we roughly know the time constant of the neuron we can use the slope of the initial change to extrapolate how the membrane potential would have changed (*dashed black line*), had not it been influenced by the above mentioned cascade (*2*). The resulting asymptotic value (*right green filled circle*) is an estimation of the background input (*3*). Since the estimation is based on the slope shortly after the inhibition onset it is an estimation of the background input at that time point (*left green filled circle*). **(E)** The background (*green*) and specific (*blue*) input should be as complementary as possible. In other words the specific input should represent those signals, and only those signals, that are not represented in the background input. For example, if the specific input has been estimated by recording the activity of neurons that project to the target area, then only those projecting neurons should be inhibited (*middle*); it would be suboptimal to inhibit all neurons irrespective of if they project or not, since this make the inhibition more unselective than the recording of the specific input (*left*), or it would be suboptimal to inhibit only the axons of the projecting neurons in the target area since this makes the inhibition more selective than the recording of the specific input (*right*).

A neural cell in the brain is submerged into a heterogeneous input field. Neural cells are squeezed between other cells which are pushing, electrifying, feeding, starving, sedating and tickling them. This hetereogenous input works at different timescales and is governed by a range of cells such as astrocytes, neurons and the chemical surrounding of a neuron. The response of any of those cells will be distributed across the whole brain to maintain the complex input field. The result is an extraordinary ongoing dynamics which has the potential to be far from linear. So how do we study the brain? If we put in an electrode we can record the output but we cannot isolate which input was responsible for the output. Although perturbations allow us to “play in” and therefore to isolate the effect of a certain signal, the ever remaining question will be if the perturbation was biologically plausible and/or if it disrupted the balance of the circuit (Buzsáki and Schomburg, 2015). Therefore we need ways to separate input signals in terms of the natural ongoing activity in the brain. We stress that for verifying the importance of a specific input signal to a neuronal population it is not enough to show that it can explain the resulting population activity. It is equally important to show that this activity cannot be explained by the activity caused by the remaining input signals. This remaining input will from now on be referred to the background input. Thus, we suggest to separate the natural ongoing input to each neuron into a background input and a specific input (Figure 1A). Those two inputs will generate the total activity in the target neuron or population. In the next section we summarize the experimental constraints for dividing the total input into those two signals.

## Dividing the Total Input to a Neuron Into a Background and a Specific Input

Using the drug curare, the specific input from the electric organ discharge (EOD) can be eliminated from the activity of the electro receptors in the electric fish (Russell and Bell, 1978; Bell, 1981). The remaining input (i.e., background input) is the efference copy signal. This signal separation gave a rough understanding of the different input signals and an orientation towards the next necessary experiment. Several years later the efference copy signal was directly recorded in the granule cells which in turn led to the development of a rigorous, data-driven model of sensorimotor integration (Kennedy et al., 2014). This example shows how the separation of ongoing brain signals allows for the deciphering of internally driven activity; the activity is dictated by the fish itself and cannot be controlled by clever sensory stimulation by the researcher. To base future experiment on the internally driven activity may be even more fruitful for the mammalian brain since it can think and plan to a larger extent, even without receiving sensory input and without causing motor output.

Curare is a potent drug that silenced the EOD activity completely. A newer way to inhibit activity is to use opsins. Although opsins have many advantages over pharmacological approaches they are dependent on the virus expression, and they may not result in a complete inhibition. If the inhibition is incomplete it will most likely move spikes in time such that an eventual spike timing code will be disturbed. Furthermore, if the inhibition is incomplete it means that the background signal may not be estimated correctly. The background signal is especially important if one wants to differentiate between a linear and non-linear operation between specific and background input since the effect of the specific input on the target activity is independent on the background input in the linear case, whereas it is dependent on it for the non-linear case. Therefore it is advantageous to also record the specific input during the inhibition in order to quantify how efficient inhibition is (Figure 1B).

Inhibition speed is another important parameter when estimating the background input (Figure 1C). When we inhibit the specific signal we will remove its contribution to the target activity. The remaining activity is an estimation of the background input. Ideally the inhibition of the specific input should be so fast that the background input cannot react to the inhibition. In other words the estimated background input reflects the natural ongoing background input until it becomes influenced by the inhibition. Although brain processes have a multitude of overlapping time constants there seems to be a possibility to record natural ongoing background input for around 5 ms after inhibition onset (Eriksson, 2016a). During this time astrocytes, inter-areal connections, vasculature and extracellular ionic signals contribute with their natural ongoing activity (Figure 1A). Afterwards some of those signals becomes influenced by the inhibition and they no longer represent the natural ongoing input. Inputs that react faster are covered by fast chemical synapses, gap junctions and ephaptic effects and those must be recorded locally with for example dense extracellular recordings and calcium imaging methods. In general, because of the growing distortion of the ongoing activity after the inhibition the background input estimation ought to be based on the target activity immediately after inhibition onset (Figure 1D). The optimal duration of this estimation (i.e., optimal inhibition duration) can be calculated with a general formula that also takes additional recordings of recurrent and indirect pathways into account (Eriksson, 2016a).

Just before the specific input is inhibited we should record it. The specific input can be estimated in multiple ways (Eriksson, 2016b). The coarsest way is to approximate the specific input by unselective recordings in the source population in which only a subset of the neurons are projecting to the target population. A middle way is to record from only those neurons that project to the target neurons. The most selective way is to record the activity directly at the synapses that constitute the specific input. Which of those three ways is preferable depends on how the inhibition is done. For example, an unselective inhibition should be matched by an unselective recording of the specific signal. The goal should be to inhibit, and record from, the same specific input (Figure 1E). This allows the specific input to “fill in” what has been inhibited during the background input estimation. As a result all input signals to the target population have been covered.

## Describing and Verifying Existing Brain Schemes

Here we will discuss how common brain mechanisms can be described, and potentially verified, without sensory and behavioral reference, using ongoing activity only. To this end we will use three signal types: the background input, the specific input and the target activity. We will cover various aspects of brain function such as linear and non-linear input integration, dynamic gating, recurrent networks, inhibitory circuits and plasticity.

**Figure 2:**
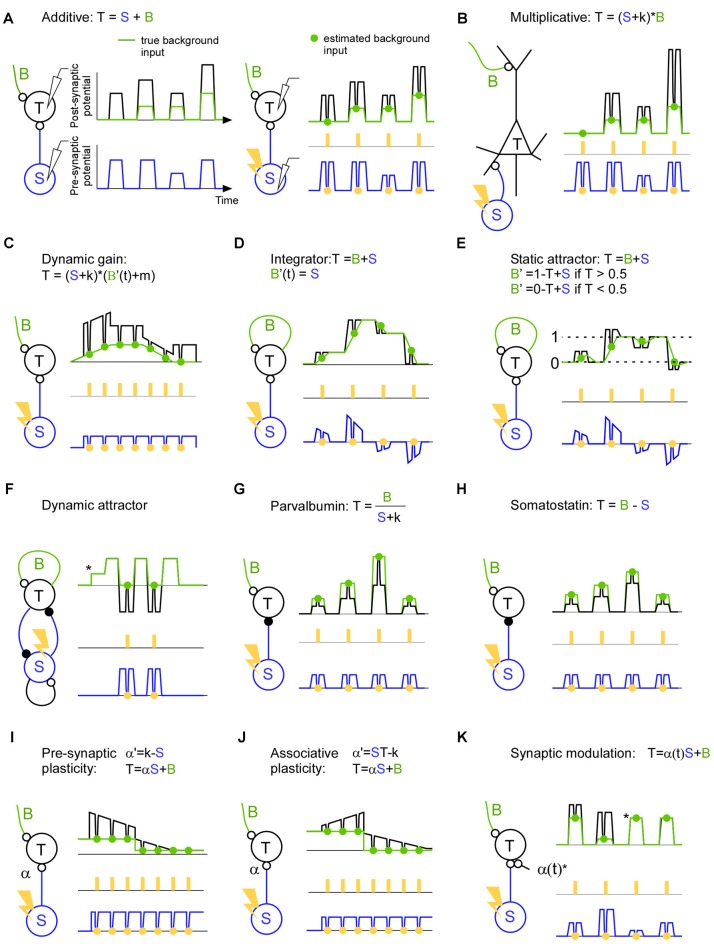
**A description of brain mechanisms using a specific (S), a background (B) and a total signal (T). (A)** *Left*: in this hypothetical example the target activity (*black line*) is the sum of the true background input (*green line*) and the specific input (*blue line*). Both the background input and specific input show simultaneous step function-like increases and decreases. This synchronicity illustrates a worst case scenario since it becomes non-trivial to separate the two input signals. *Right*: estimation of the background input. The specific input is inhibited (*orange points*) with a light pulse (*orange rectangles*) in order to estimate the background input (*green points*). The inhibition causes a trough in both the target activity and specific input. Note that the trough in the specific input is proportional (equal in this hypothetical example) to the trough in the target activity indicating a linear summation of background and specific input. **(B)** The target activity is the result of a multiplication between the background and the specific input. Note that in contrast to the linear case described in panel **(A)**, the trough in the specific input is not proportional to the trough in the target activity indicating a non-linear summation of background and specific input. **(C)** The target activity is the result of a dynamic gain mechanism which amplifies the specific input if the background input increases. Note that the background envelope might be a piece of an oscillation and that this oscillation may be so quick that only one light pulse can be delivered per phase. In this case the effect of the inhibition has to be related to the phase of the target activity since that can be recorded continuously up to the point of the inhibition. **(D)** The target activity is the specific input integrated over time. Recurrent connections sustains/remembers the target activity such that a new specific input will be added on top of the previous target activity. The background input describes the contribution from the recurrent connections. The negative specific input is shown for illustrative purposes and can be implemented using feedforward inhibition or competition through inhibition. Note that each light pulse (*orange*) should be seen as an individual trial; in the case of an integrating mechanism it is advantageous if the light pulses are not coming in close succession since the inhibition itself will change the integration. **(E)** Attractor network with two attractors (*dashed line 0* and *dashed line 1*). If the specific input becomes similar to that of an attractor (*dashed line 0* or *dashed line 1*) the background activity increases and pulls the target activity towards the attractor. **(F)** The target activity in a dynamic attractor changes over time. Once the target neuron has received an input (*) the activity starts to oscillate. In the example an excitatory (small open circle denotes the synapse) and inhibitory (small filled circle denotes the synapse) neuron is reciprocally connected to implement an oscillator. By estimating the background input we can see which part of the oscillation is due to the inhibitory neuron, and we can see that there is no other inhibitory source that gives rise to the oscillation. **(G)** The target activity is the background input divided by the specific input. **(H)** The target activity is the background input minus the specific input. **(I)** The target activity is the result of the specific input times the synaptic strength (α) plus the background input. In this example the synapse is a depressing one which decreases the efficacy when it is used. **(J)** Like in **(I)** but for associative plasticity in which the efficacy of the synapse is increasing when both the pre- and post-synaptic activity is high, and in which the efficacy is decreasing when one of the pre- and postsynaptic neuron has low activity. **(K)** The target activity is the sum of the background input and the specific input that runs across synapse (α(t)) that blocks the input during the third and the fourth pulse (*).

An example of a linear mechanism is when a neuron responds according to the sum of its inputs (Figure 2A). In an elegant study, such a linearity could be determined at the onset of a sensory response since the underlying ongoing activity during the sensory response onset could be predicted from the ongoing activity before the sensory response onset (Arieli et al., 1996). Through the use of short lasting inhibitions of the specific thalamo-cortical input it may be possible to extract the ongoing background input at any time point during a sensory stimulation, and not only during its onset. Moreover if we record the specific thalamo-cortical input shortly before it was inhibited we can understand how ongoing fluctuations of the thalamic activity is interacting with ongoing fluctuations of the ongoing cortical activity on a single trial basis. Such a trial resolved separation may also be important in order to understand responses to unexpected stimuli in the framework of error and predictive coding (Rao and Ballard, 1999; Friston, 2010). Another linear model describes how the coherence between two oscillating populations depends on the communication strength (k) and delay (Δ*t*); *T = B*_amplitude_ sin(f*t+B_phase_) + *S*_amplitude_ k sin(f*t+S_phase_+Δ*t*), where *f* is the frequency of the oscillation, *t* is time, *T* is the target activity, *B* is the background input, and *S* is the specific input (Eriksson et al., 2011). This model shows that if both the background and specific input are taken into account it is possible to generate phase dependent power correlations that are indistinguishable from that of the experimental results, even when there is a connection delay (Womelsdorf et al., 2007).

A classical example of a non-linearity is that of the pyramidal cell (Larkum et al., 1999, 2004; Figure 2B). Such a mechanism may contribute to a multiplicative interaction between sensory and spontaneous activity (Haider and McCormick, 2009; Reig et al., 2015). In fact for Bayesian coding it is assumed that sensory activity is modulated by expectations (Lee and Mumford, 2003; Saleem et al., 2013). Such a modulation may also be used to direct the flow of information in the brain during attention for instance. If the neurons that send the specific input are synchronized they may transmit the message more effectively to the target population (Jia et al., 2013; Zandvakili and Kohn, 2015). A control of those results is to show that the increased transmission cannot be explained by another pathway; in other words there is no modulation in the background input during the same time. Nevertheless, the increased communication may lead to a higher coherence between the source and the target area. This increased coherence may in turn govern the communication through coherence theory in order to sustain the communication over time (Fries, 2005, 2015). Indeed, action potentials are gated if they arrive on a certain phase of an artificial oscillation in the target structure (Cardin et al., 2009; Siegle et al., 2014; Ni et al., 2016). Although this background input is crucial for describing the dynamic gating it has so far been overlooked (Figure 2C). Much can be discovered regarding neuronal communication if we record the specific and the target signals (Buzsáki and Schomburg, 2015); however, if we do not record the background input, we will be blind to various false positives. This is because the “hidden” background input can mask how the target activity responds to the specific input.

Recurrent networks are thought to generate everything from oscillations to complex ongoing activity. The simplest form of a recurrent network can integrate the input across time (Figure 2D). For example, the input can convey the evidence for performing a certain action. Such a mechanism may be central in decision making (Huk and Shadlen, 2005; Shadlen and Shohamy, 2016). The longer the network integrate the information the easier it may be to make a decision. Here, the background input is the integrating signal. Attractor networks, on the other hand, are partly driven by the input, but as soon the input gets close to a stored memory the background input takes over and drives the activity to that memory (Hopfield, 1982; Fransén and Lansner, 1998; Kaplan and Lansner, 2014; Wimmer et al., 2014; Figure 2E). Finally, there are attractors that are dynamic in the sense that they generate activity that does not settle to a constant activity (Figure 2F). Such networks can store and recall sequences for motor behavior and memory sequences (Churchland et al., 2010; Sussillo et al., 2015). They can also implement functions such as an oscillator with an excitatory and inhibitory neuron (Whittington et al., 2000). Here the background input can be the excitatory inputs that initiate the cycle and the specific input can be the input that ends the cycle such that the cycle can start anew.

With the introduction of optogenetics it has been possible to identify and perturb specific cell types such as parvalbumin and somatostatin expressing inhibitory neurons (Lima et al., 2009). Although much have been learned from their qualitative behavior and qualitative influence on target cells (Atallah et al., 2012, 2014; Lee et al., 2012, 2014; Wilson et al., 2012; El-Boustani et al., 2014), it remains to be shown how they influence the target cells during ongoing activity. During animal behavior they will not only perform additive or subtractive normalization (Figures 2G,H), and they will not only be involved in slow and fast oscillations (Buzsáki and Wang, 2012). They will probably do a combination if not more (Kepecs and Fishell, 2014). For understanding their function it is important to know which background activity their inhibition meets. After all, the role of an inhibitory neuron must be to modify some existing background activity such that the resulting activity governs brain function.

Synaptic efficacy and plasticity can be directly assessed using the background and specific input. Since the specific input quantifies the signal that has been sent from the pre-synaptic neuron and the inhibition will tell how much of that signal was continuing to the post-synaptic cell, we can quantify the efficacy of the synapse changes using natural ongoing activity. For pre-synaptic plasticity the influence of the input can increase or decrease over time dependent on the pre-synaptic activity (Markram and Tsodyks, 1996; Figure 2I). For associative plasticity the efficiency of the synapse is increasing if both the input and target activity is high, whereas the efficiency is decreasing if the input or the target activity is low (Hebb, 1949; Figure 2J). Here it may be interesting to know the background input since it can pre-depolarize the target neuron before the specific input arrives, and hence remove the magnesium block necessary to induce long term memory (Cull-Candy and Usowicz, 1987; Jahr and Stevens, 1987; Ascher and Nowak, 1988). Finally, for the so-called tripartite synapse a third cell can control the strength of the synapse (Figure 2K). This third cell can for example be a local astrocyte, cholinergic or dopamine neuron (Kimura et al., 1999; Perea et al., 2009; Tritsch and Sabatini, 2012; Allen, 2014). The resulting network reorganization would be crucial for anapoietic adaptive mechanisms (Nikolić, 2015).

In all those examples we have dealt with one-dimensional signals. For an experimental setting in which multiple neurons are recorded each of those signals will be multidimensional. The mapping from the multidimensional background and specific input to the resulting multidimensional target activity can be made with general purpose dimension reduction and mapping techniques (Hinton and Salakhutdinov, 2006; Cireşan et al., 2010). To be able to understand how a brain scheme is implemented in a multidimensional manner is important since activity differences across neurons code information for everything from sensory events to motor commands (Georgopoulos et al., 1982; Serre et al., 2007; Elsayed et al., 2016). To make things extra complicated, a neuron uses its temporal activity profile to code information, and different neurons have different temporal profiles (Richmond et al., 1987; Elsayed et al., 2016). Therefore when we excite a population of neurons unselectively, the brain may inhibit the response since the population activity may not mean anything to the brain (Logothetis et al., 2010). Although it recently became possible to artificially stimulate dozens of cells it is not yet possible to stimulate a whole brain area with a realistic activity pattern (Rickgauer et al., 2014; Packer et al., 2015; Chaigneau et al., 2016). Therefore, we are proposing a non-excitation approach, in which the sole purpose of the inhibitory perturbation is to measure a neuron specific ongoing background input signal. The artificial stimulation methods on the other hand will be instrumental for detecting connections between cells in order to estimate the inter-cellular specific input.

## Conclusion

Here we have described one way to approach many brain mechanisms ranging from dynamic attractor models to plasticity. The list of schemes with one specific input signal can be made very long because it is natural for a researcher to focus on one specific signal while lumping all remaining signals into a “background signal”. Nevertheless for multi-input theories the approach is scalable since signals from additional neuronal populations can be included in the specific input. To estimate the background input for this case, all those additional populations should be inhibited. In the future when we have a theory of how all signals are integrated, and we can measure the signals from all presynaptic cells, from all extracellular ionic signals, and from all astrocytes there will be no background input and no need to inhibit.

The division of background input and specific input on a single trial basis will allow the researcher to study brain derived activity with the same ease as sensory derived activity. Noise correlations could then be studied with the same level of control as signal correlations can be done today. Up to now researchers have largely relied on sensory and behavioral control of neuronal inputs. For example a mouse can be trained to walk with a certain speed meanwhile it is stimulated with a drifting grating that induces the visual illusion of movement (Keller et al., 2012; Saleem et al., 2013). Since the walking speed and drifting speed can be controlled by the experimenter one can see how those variables are integrated in the recorded neurons. In this case the ongoing activity is averaged out such that one can focus on sensory and motor related variables. This is not to say that ongoing activity is unimportant. In contrast, the interaction between sensory and ongoing activity is probably fundamental to how we perceive our environment. Furthermore, in non-sensory areas such as frontal and motor areas the ongoing activity may be relatively stronger since it reflects the thinking and intention of the animal. In such areas it would be crucial to define the input in terms of ongoing activity since the stimulus derived activity is weaker. As a result it may be possible to understand how the spontaneous activity in different areas or populations are integrated to facilitate new intentions, planning and behavior. Nevertheless, the success of this approach relies on strong and fast inhibition and the measurement of inter-cellular signals.

## Author Contributions

The author conceived and performed the study.

## Funding

The article processing charge was funded by the German Research Foundation (DFG) and the University of Freiburg in the funding programme Open Access Publishing.

## Conflict of Interest Statement

The author declares that the research was conducted in the absence of any commercial or financial relationships that could be construed as a potential conflict of interest.
